# Orchard Microclimate Control as a Way to Prevent Kiwifruit Decline Syndrome Onset

**DOI:** 10.3390/plants14071049

**Published:** 2025-03-28

**Authors:** Claudio Mandalà, Francesco Palazzi, Grazia Federica Bencresciuto, Carmela Anna Migliori, Cristina Morabito, Chiara Morone, Luca Nari, Stefano Monaco, Laura Bardi

**Affiliations:** 1 CREA Consiglio per la Ricerca in Agricoltura e l’Analisi dell’Economia Agraria, Research Centre for Engineering and Agro-Food Processing, 10135 Turin, Italy; claudio.mandala@crea.gov.it (C.M.); francesco.palazzi@crea.gov.it (F.P.); graziafederica.bencresciuto@crea.gov.it (G.F.B.); carmelaanna.migliori@crea.gov.it (C.A.M.); stefano.monaco@crea.gov.it (S.M.); 2 Department of Agriculture, Forest and Food Sciences, University of Turin, Largo Paolo Braccini 2, 10095 Grugliasco, Italy; cristina.morabito@unito.it; 3 Phytosanitary and Scientific-Technical Services Department, Agricultural and Food Directorate, Piedmont Region, 10144 Turin, Italy; chiara.morone@regione.piemonte.it; 4 AGRION, The Foundation for Research, Innovation and Technological Development of Piedmont Agriculture, 12030 Manta, Italy; l.nari@agrion.it

**Keywords:** climate change, abiotic stress, vapor pressure deficit, over-tree irrigation, shading net, leaf temperature, leaf gas exchanges, stem water potential, xylem vessels, hydraulic conductance

## Abstract

A syndrome called “Kiwifruit Decline Syndrome” (KiDS) affects kiwifruit in several Mediterranean areas, causing growth arrest and wilt that rapidly progress to desiccation, scarce root growth, absence of fibrous roots, brown soft-rotting areas, and cortical detachment from the central cylinder. The origin is considered multifactorial, and a correlation with hydraulic conductance impairment caused by a high vapor pressure deficit (VPD) and temperature was detected. In this work, over-tree micro-sprinkler irrigation and shading nets were tested to protect leaves from overheating and locally decrease VPD. Leaf gas exchanges, leaf temperature, stem water potential, stem growth, root starch content, root xylem vessel diameter, density, and vulnerability to cavitation were assessed. A positive effect of over-tree irrigation associated with shading was observed: lower leaf temperature, higher stem water potential, stomatal conductance, and photosynthesis were detected; moreover, root starch content was higher in the summer. Narrow xylem vessel diameters were observed, indicating a long-term adaptation to rising VPD for lower vulnerability to cavitation, in all plants, but higher diameter, lower density, and higher vulnerability index indicated lower plant water stress under over-tree irrigation associated with shading. These results indicate that microclimate control by proper agronomic management can protect kiwifruit from climate stress, decreasing the risk of KiDS onset.

## 1. Introduction

Kiwifruit is a woody liana native to China, where nowadays, it is extensively cultivated, even if its fruits are still currently collected from the wild. It is a highly value-added crop with high market demand and characteristics that have favored its spread in other countries. At present, the worldwide production is about 4,500,000 tons per year [1], which is mainly located in China, New Zealand, Italy, Greece, and Chile; 50% of totally produced and 90% of internationally traded kiwifruits are from the green pulp variety Hayward (*Actinidia chinensis* var. *deliciosa*) [2].

Kiwifruit prefers a cold climate and high altitudes, and it needs adequate winter chilling; it grows spontaneously in humid and shaded places and escapes the wind. It is characterized by numerous very large leaves, long and flexible vines, and very wide xylem vessels, which confer remarkable sensitivity to drought. Moreover, it has an anisohydric behavior, so when drought stress occurs, it maintains stomata opening, and leaf water potential can drop to lower levels in comparison to isohydric plants: this allows for the maintenance of high levels of photosynthetic activity, but also of transpiration, so severe dehydration can happen under prolonged water unavailability [3,4,5]. Due to its commercial interest, its cultivation has spread in regions where the environmental and climatic features are far from those of its natural habitat. In several regions where the kiwifruit is widely cultivated, climate change has further steadily worsened the stress induced on plant physiology by unfavorable conditions [2,6].

High temperatures in kiwifruit plants reduce fruit carbohydrate accumulation, hinder root growth and starch accumulation, and impact flowering and growth in the following season [7,8,9] due to the shift in photosynthate allocation towards vegetative growth [10]. High temperatures also reduce soil oxygen, creating additional stress for the roots that are highly sensitive to hypoxic soil conditions [7,8,9,11,12,13]. High temperature, drought stress, and vapor pressure deficit (VPD) cause xylem hydraulic conductance failure due to xylem vessel cavitation and implosion; moreover, hydraulic conductance is also reduced by the narrowing of xylem vessels, a long-term adaptation strategy adopted to prevent cavitation [14,15]. Starch unavailability in the xylem parenchyma may significantly affect the ability to restore the hydraulic conductivity of cavitated xylem vessels by refilling through starch hydrolysis [16,17].

Climate change can also favor the occurrence of biotic threats [2,18,19,20,21,22], and the interplay of biotic and abiotic stress can severely affect the responsiveness and survivability of the plant: this is supposed to happen in the case of kiwifruit decline syndrome (KiDS). KiDS was indicated as a new syndrome in 2012 in Italy in northern regions; then, it spread throughout the whole Italian territory, causing, at present, the uprooting of wide areas of kiwifruit orchards and a decrease of about 30% of the production [23]. The KiDS symptoms arise in the root system and the canopy. In the root system, the damages are scarcity or absence of the fibrous roots, structural roots showing red discoloration under the cortex, phloem detachment from the central stele, and breakdown of the cortex. In the canopy, the damages appearing progressively are leaf desiccation starting from the margin, leaf deformation and sprout desiccation, stunted fruit growth and wide fruit drop, widespread phylloptosis, and plant collapse; generally, affected plants die within 2 years [24]. Currently, there are no effective ways to treat or prevent this issue.

Agronomic management could have a major role in preventing plant environmental stress by reproducing as best as possible the conditions of the natural habitat. Irrigation systems, mulching, soil conservation tillage methods, cover cropping, and shade management can address issues arising due to climate change [2].

Water management is particularly troublesome in kiwifruit cultivation, due to simultaneous high water demand and high sensitivity to waterlogging; consequently, precision irrigation is necessary, based on soil moisture detection, to assess the irrigation volumes [23,25]. Drip irrigation has widely spread following the KiDS onset as a way to limit the risk of waterlogging and to reduce soil-borne diseases, but this practice did not prevent KiDS progression.

Over-tree micro-sprinkler irrigation is another possible way to relieve climate stress by decreasing leaf temperature and VPD [26,27]. This practice could allow leaf homeostasis and protection from overheating at once with water delivery: this could be particularly effective during hot summer days, when plant hydraulic conductance is hindered, and leaf temperature can exceed threshold values for photosynthetic activity and even for survival [15].

Also, shade nets can have a role in improving the crop’s microclimate by protecting plants from excessive radiation, high temperature, and drought stress: anti-hail nets can decrease leaf temperature and VPD and root zone temperature, reducing crop water requirement and improving water-use efficiency [28]. Plants in kiwifruit orchards are fully sun-exposed, even if they prefer shady conditions in their natural habitat. The effect of shading in kiwifruit cultivation has been studied in relation to fruit yields and quality [29,30,31] and to plant physiological response [32], with different results based on net materials and spectral properties of photo-selective nets.

It can be hypothesized that suitable agronomic management could contribute to preventing KiDS onset by controlling the kiwifruit orchard microclimate in order to counteract the stresses induced by climate change. In this work, over-tree micro-sprinkler irrigation was assessed, associated with shading nets, as a microclimate control system aimed at protecting leaves from overheating and at decreasing vapor pressure deficit stress at the canopy level. Plant biometric and physiological parameters were detected for two years; roots were also analyzed in order to evaluate the starch content and the xylem functionality by checking the xylem vessel morphology.

## 2. Materials and Methods

### 2.1. Study Site and Trial Setup

**Figure 1:**
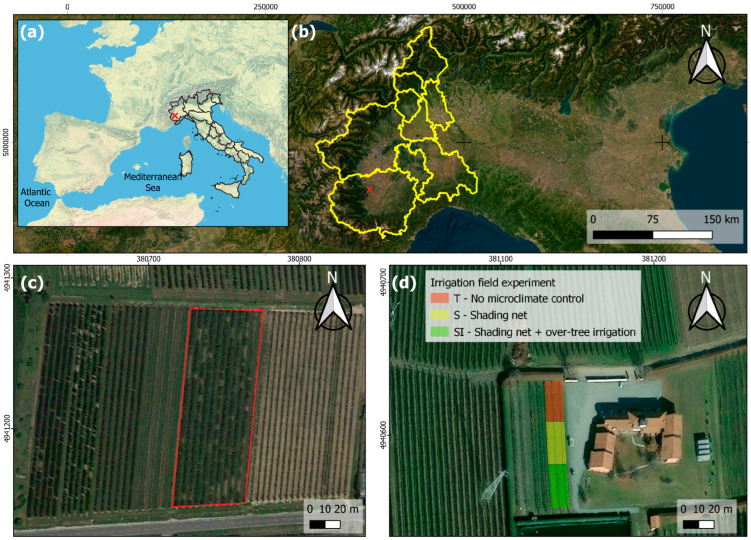
Localization of the study site: (**a**) map of Italy (red cross: experimental orchard location); (**b**) map of Piedmont (red cross: experimental orchard location); (**c**) a KiDS-affected orchard (red box) located nearby the experimental orchard: wide failures are evidence of uprooting following plant death due to KiDS; (**d**) experimental orchard.

The study was carried out in 2022 and 2023 in a new planting experimental orchard located in the province of Cuneo in Piedmont (Northern Italy) (Figure 1), in an area where KiDS caused the uprooting of about 40% of kiwifruit orchards over the last 10 years. According to the Köppen–Geiger classification [33,34], the area is characterized by a Cfa (i.e., warm temperate) climate, without a dry season and with a hot summer. In the investigated site, the soil is classified as Dystric Fluventic Eutrudept, coarse-loamy, mixed, nonacid, mesic [35], or Fluvic Cambisols [36].

The experimental trial was very close to other orchards affected by KiDS. The location and main characteristics of the orchard are reported in Table 1.

The orchard was divided into three blocks and designed perpendicularly into rows. Each treatment corresponded to two rows for a total of 40 plants per treatment. Four plants were randomly selected inside each block, in order to have four replicates, to perform physiological, anatomical, and biometric analyses using leaf gas exchanges, leaf temperature, stem water potential, stem growth, root starch content, root xylem vessel diameter and density, and vulnerability to cavitation. The treatments compared were (i) no microclimate control (T); (ii) shading by a black-yellow anti-hail shading net (Iridium Dual B^®^, Agrintech srl, Eboli, Italy; material: HDPE, 17–21% shading) associated with over-tree micro-sprinkler irrigation (SI); and (iii) shading by a black-yellow anti-hail shading net (S). Over-tree irrigation was carried out from mid-June to the end of August, three times per day (10:30–12:30–14:30), seven minutes each, with a fixed total daily irrigation volume of 6.9 m^3^ ha^−1^. The orchard management was carried out according to usual local agronomical practices. All treatments were irrigated by drip irrigation with watering volumes calculated to restore soil water depletion by monitoring the soil moisture at two depths (20 cm and 40 cm) with soil moisture sensors (Watermark, Irrometer Company Inc., Riverside, CA, USA); the total seasonal irrigation volume of drip irrigation was 607 m^3^ ha^−1^ in 2022 and 430 m^3^ ha^−1^ in 2023.

### 2.2. Environmental Measurements: Weather Data, Photosynthetically Active Radiation (PAR), and Microclimate

Weather data were collected from an agrometeorological station (METOS^®^ station, Pessl Instruments GmbH, Weiz, Austria) located in the experimental site. Weather data collected included precipitation (mm), air temperature (°C), solar radiation (W m^−2^), relative humidity (%), wind speed (m s^−1^), and vapor pressure deficit (VPD, kPa).

In T and S (under the net) treatments during 2022, the full-spectrum (400–700 nm) PAR (µmol m^−2^ s^−1^ Photosynthetic Photon Flux Density, PPFD) was monitored by sensors like Apogee FullSpectrum SQ-500-SS (Apogee Instruments, Inc., North Logan, UT, USA), and relative humidity (RH, %) and air temperature (°C) were monitored by Tinytag Ultra 2—TGU-4500 data loggers (Gemini Data Loggers Ltd., Chichester, West Sussex, UK)

### 2.3. Physiological and Biometric Measurements

#### 2.3.1. Leaf Gas Exchanges

The measurements were carried out during 2022 and 2023. During 2022, the measurements were carried out at three different times during the day, in the morning (09:30 a.m.), during the central hours (1:30 p.m.), and in the afternoon (4:30 p.m.), and on three different days of the years (DOY) for three consecutive weeks during summer (DOY 209, 215, 221). During 2023, the measurements were carried out in the morning on three different days (DOY 136, 172, and 286): (i) 31 days before the starting of the over-tree irrigation treatment, (ii) 1 week after the starting of the over-tree irrigation treatment, and (iii) 28 days after the end of the over-tree irrigation treatment. All measurements were carried out on three leaves (young and fully expanded) per plant. Net CO_2_ assimilation (A, µmol CO_2_ m^−2^ s^−1^), stomatal conductance (gs, mol H_2_O m^−2^ s^−1^), and transpiration (E, mmol H_2_O m^−2^ s^−1^) were measured by an InfraRed Gas Analyser (IRGA, Portable Photosynthesis system Li-Cor, Ecosearch, Montone (PG), Italy).

#### 2.3.2. Leaf Temperature

Leaf temperature was monitored in treatments T and SI by LT-1T sensors, and the data were logged using the data-logging system SDI-12 Standard (version 1.3) (Edaphic Scientific, Melbourne, VIC, Australia) from DOY 167 to 272; the sensors were placed on one leaf per plant in four plants per treatment.

#### 2.3.3. Stem Water Potential

Stem water potential (SWP, MPa) was measured during 2023 (DOY 172 and 286, between 1:00 and 2:30 p.m.) using a pressure chamber (Ecosearch s.r.l., Montone (PG), Italy) according to [37] on two leaves per plant. Leaves were previously covered with aluminum bags for 30 min to reach a balance between leaf and stem water potentials. It was not possible to measure SWP, as it is a destructive analysis, in 2022 due to the small number of leaves in the one-year-old plants.

#### 2.3.4. Stem Growth

Stem growth was evaluated by measuring the collar diameter with a caliper in 2022 at DOY 136 and 280 and in 2023 at DOY 179 and 300 in 15 plants per treatment. The diameter increase was calculated as the difference between sequential measurements.

### 2.4. Root Xylem Vessel Characterization

Structural roots were sampled in 2023 at DOY 172 and 286 at a 20 cm depth and a 20 cm distance from the trunk and processed as previously described [15]. Sections measuring 20 μm were obtained using a Leitz 1516 rotary microtome (Leica, Wetzlar, Germany). Light microscopy analysis was carried out by a Leica DM2000 LED light microscope (Leica, Wetzlar, Germany) equipped with a digital camera (Leica DFC450C). Images were analyzed by Leica LASX 5.0.3.2480 software and processed by ImageJ software (version 1.53j) in order to measure the xylem vessel diameters (D, μm) and density (VD, vessel number mm^−2^). The vulnerability index to cavitation (VI) was calculated according to [38].

### 2.5. Root Starch Content

Root sections were prepared as described in Section 2.4 and then stained with Lugol’s iodine solution (5% iodine, 10% potassium iodide, and bi-demineralized water) according to [39]. Light microscopy analysis and image acquisition and processing were carried out as described in Section 2.4, and the stained starch area was calculated as a percentage of the total area of the section. The root sampled at DOY 286 was used to analyze the total root starch content as mg g^−1^ (dry weight, DW). Sample preparation was performed according to [40]; collected root samples were dried at 65 °C for 72 h; dry root samples were ground to a fine powder using a tissue lyser system (TissueLyser II, Qiagen, Milan, Italy); and starch content was quantified as detailed in [41]. Starch concentration was assessed using an enzymatic-based assay (STA-20 kit; Sigma-Aldrich, Milan, Italy), following the manufacturer’s instructions. Starch content was represented by the amount of released glucose, which was determined by a colorimetric reaction using a glucose oxidase-mediated method. Sample absorbance was read at 540 nm by P9 spectrophotometer (VWR, Milan, Italy), starch concentrations were calculated using the glucose standard curve as a reference and expressed as mg g^−1^ of dry root.

### 2.6. Statistical Analysis

Data were processed and analyzed by Excel (MS Software) and PAST 4.08 [42] software; ANOVA and least significant difference (LSD) (*p* ≤ 0.05) analyses were carried out. The results of the ANOVA are reported in Tables S1–S5.

## 3. Results

### 3.1. Weather Data, PAR, and Microclimate Measurements

The annual rainfall was 522 mm in 2022 and 972 mm in 2023. In Figure S1, the data of daily mean temperature and vapor pressure deficit (VPD) recorded from DOY 135 to DOY 300 during 2022 and 2023 are reported. It can be observed that both temperature and VPD were higher in 2022 than in 2023 during the summer.

Cumulated PAR measured in T and S treatments from DOY 124 to DOY 304 is shown in Figure S2: a mean of 87.4% for the relative transmittance of the yellow-black net was observed.

Relative humidity and air temperature monitored in T and S (under the net) treatments measured from DOY 124 to DOY 304 are shown in Figure S3; it can be observed that relative humidity was significantly higher and air temperature was lower in the S treatment in comparison to T.

### 3.2. Leaf Gas Exchanges

**Figure 2:**
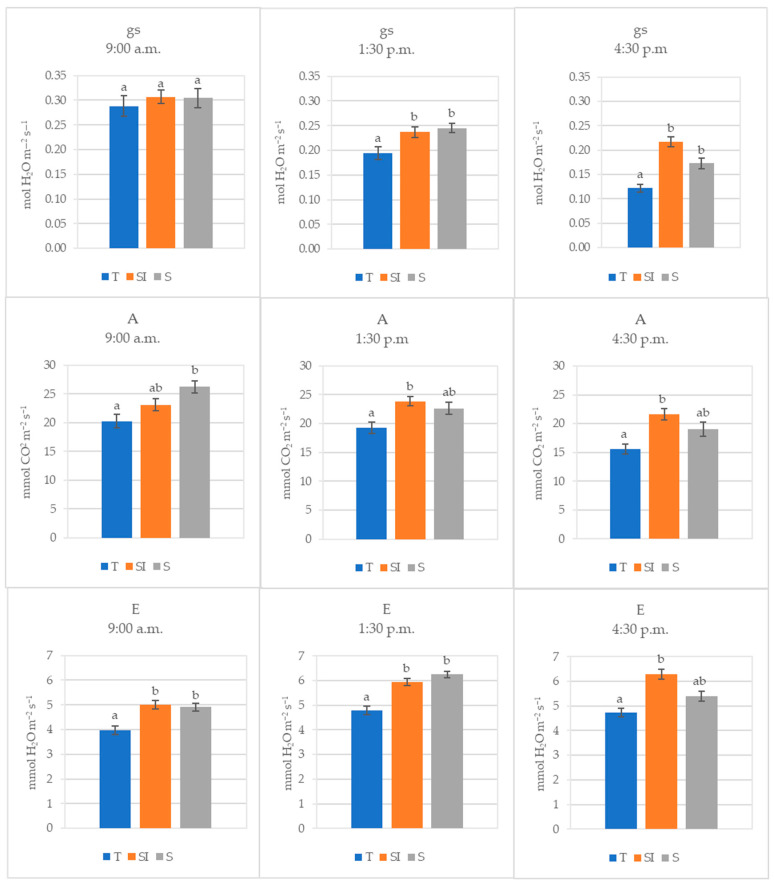
Stomatal conductance (gs, mol H_2_O m^−2^ s^−1^), net CO_2_ assimilation (A, µmol CO_2_ m^−2^ s^−1^), and transpiration (E, mmol H_2_O m^−2^ s^−1^) of T, SI, and S treatments measured in 2022 at three different times of the day (morning, midday, and afternoon). Data shown are the mean values ± standard error of detections from three leaves of four plants in three different measurements (DOY 209, 215, and 221); different letters show significant differences between the treatments according to ANOVA and least significant difference (LSD) analyses at a *p*-value threshold of ≤0.05.

The mean values of data collected during 2022 at different times during the day (morning, midday, and afternoon) are shown in Figure 2. Stomatal conductance decreased during the day, and the decrease was higher in T, where it became significantly lower than in SI and S already in the middle of the day. Transpiration was significantly higher where microclimate control was carried out, but a decrease was observed from midday to the afternoon in the S treatment, becoming equivalent to T. Net CO_2_ assimilation was significantly higher in the morning in S than in T, but it decreased during the day, while in SI, it remained nearly constant, becoming significantly higher than in T.

**Figure 3:**
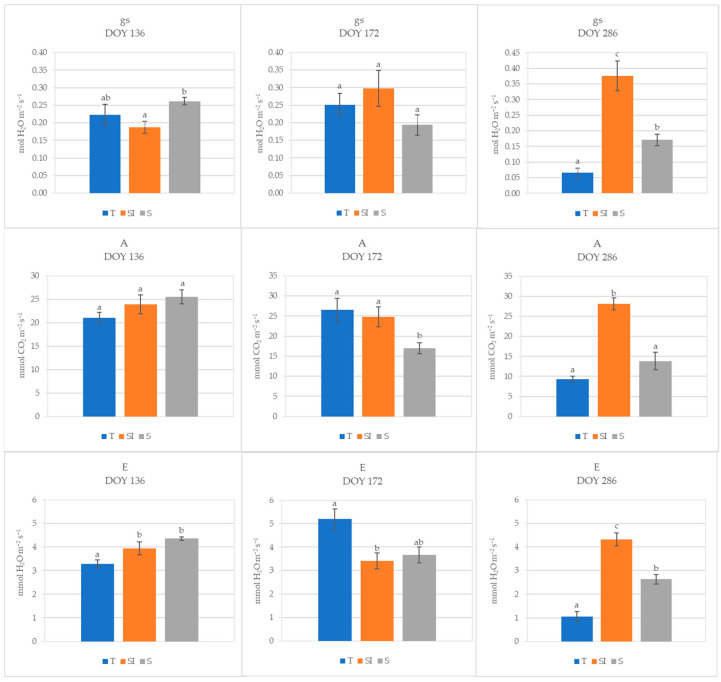
Stomatal conductance (gs, mol H_2_O m^−2^ s^−1^), net CO_2_ assimilation (A, µmol CO_2_ m^−2^ s^−1^), and transpiration (E, mmol H_2_O m^−2^ s^−1^) of T, SI, and S treatments measured in 2023 at three different times (DOY 136, 172, and 286). Data shown are the mean values ± standard error of detections from three leaves of four plants; different letters show significant differences between the treatments according to ANOVA and least significant difference (LSD) analyses at a *p*-value threshold of ≤0.05.

During 2023, at DOY 136, stomatal conductance in T plants was not different from microclimate-controlling treatments, while it was significantly higher in S than in SI; no differences were detected for net CO_2_ assimilation, while transpiration was significantly lower in T than in SI and S. At DOY 172, no differences were detected for stomatal conductance among treatments, while net CO_2_ assimilation was significantly higher in T and SI than in S, and transpiration was significantly lower in SI than in T. On DOY 286, the SI treatment showed significantly higher values of stomatal conductance, transpiration, and net CO_2_ assimilation in comparison to both T and S; moreover, stomatal conductance and transpiration were significantly higher in S than in T (Figure 3).

### 3.3. Stem Water Potential (SWP)

**Figure 4:**
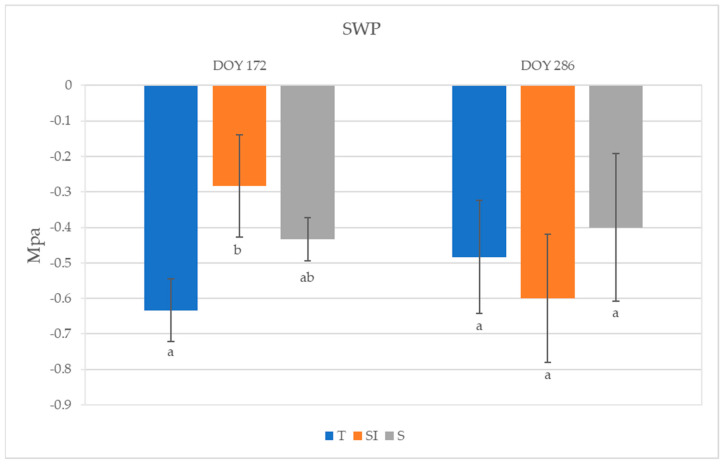
Stem water potential (SWP) of plants from T, SI, and S treatments. Data shown are the mean values ± standard error of data recorded from 3 plants per orchard in 2023 (DOY 172 and 286); different letters show significant differences between the treatments according to ANOVA and least significant difference (LSD) analyses at a *p*-value threshold of ≤0.05.

The highest SWP was observed in SI plants (−0.28 MPa) and the lowest was observed in T plants (−0.63 MPa) at DOY 172; a significant difference was detected between plants undergoing T and SI treatments (Figure 4). An SWP increase was observed from DOY 172 to DOY 286 in T and S plants, while it decreased in SI. No significant differences among treatments were detected on DOY 286.

### 3.4. Leaf Temperature

**Figure 5:**
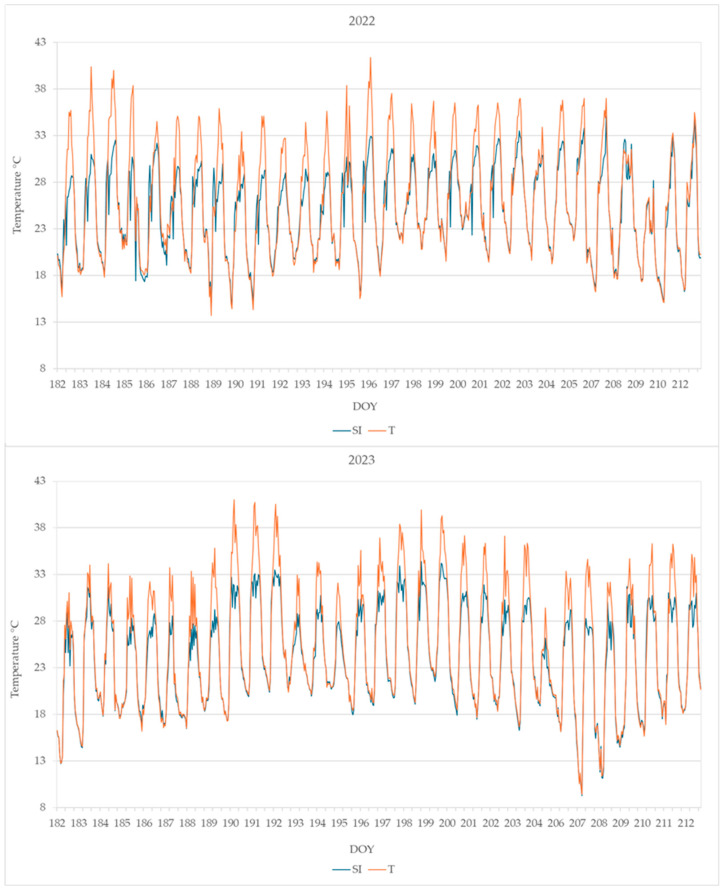
Leaf temperature monitored from DOY 182 to 212 in plants from the SI and T treatments. Data shown are the mean values of data detected from 4 plants per treatment.

The daily maximum leaf temperature was higher in plants from T than from SI; the leaf temperature detected from DOY 182 to DOY 212 is shown in Figure 5.

The highest leaf temperatures reached during the summer months (June, July, and August) are reported in Table 2. Very high temperatures were reached in treatment T, higher than 40 °C in both years 2022 and 2023, while in treatment SI, the highest leaf temperature was 35.1 °C in 2022 and 35.9 °C in 2023.

### 3.5. Stem Growth

No significant differences were observed during 2022 for stem growth. The increase in stem diameter during 2023 became significantly higher in treatment SI, where the mean increase in collar diameter from DOY 173 to DOY 300 was 5.13 cm, compared to the T treatment, where the increase was 2.24 cm.

### 3.6. Roots Xylem Vessel Characterization

**Figure 6:**
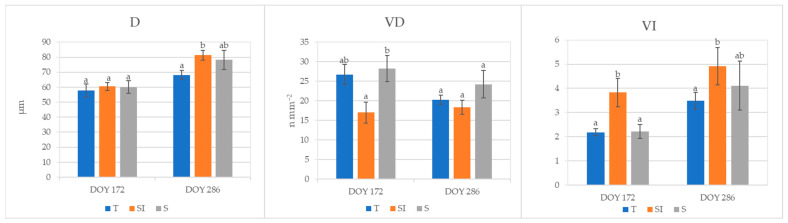
Roots xylem vessel characterization of plants from T, SI, and S treatments, carried out on samples collected in 2023 at DOY 172 and 286. Data shown are the mean values ± standard error of data detected from 8 plants per treatment; different letters show significant differences between the treatments according to ANOVA and least significant difference (LSD) analyses at a *p*-value threshold of ≤0.05. D = vessel diameter (μm); VD = vessel density (n mm^−2^); VI = vulnerability index to cavitation.

Root mean xylem vessel diameter (D, µm), xylem vessel density (VD, n mm^−2^), and vulnerability index to cavitation (VI) analyzed in root samples collected at DOY 172 and 286 are shown in Figure 6. Xylem vessel diameter was significantly higher at DOY 286 in plants from the SI treatment in comparison to T, and it rose from DOY 172 to DOY 286, varying from 57 to 68 mm in plants from treatment T and from 60 to 81 mm in plants from treatment SI. Vessel density at DOY 172 in plants from the S treatment was comparable to T plants and significantly higher in comparison to the SI treatment; it remained almost constant from DOY 172 to DOY 286 in SI, while it decreased in T and S treatments. Vulnerability index to cavitation was significantly higher in plants from SI treatments in comparison to T and S at DOY 172; it rose from DOY 172 to DOY 286 in all treatments, and at DOY 286, plants from SI treatments showed a VI that was still significantly different from T plants.

### 3.7. Root Starch Content

**Figure 7:**
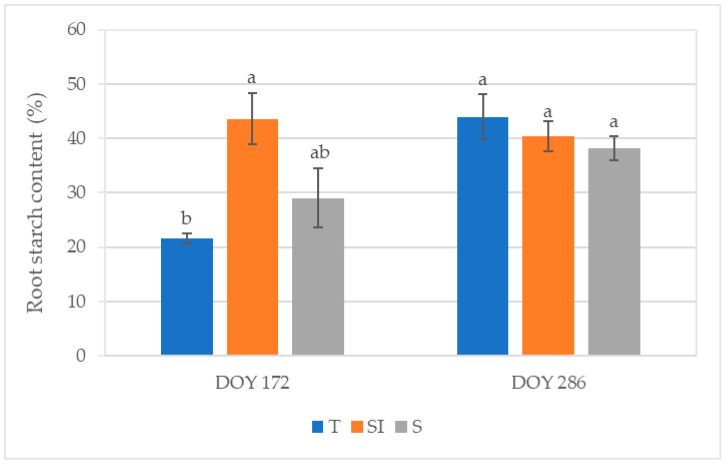
Root starch content expressed as a percentage of the Lugol-stained area of the total root sample area detected by light microscopy in root sections from samples collected at DOY 172 and DOY 286 from T, SI, and S treatments. Data shown are the mean values ± standard error of data detected from 8 plants per treatment; different letters show significant differences between the treatments according to ANOVA and least significant difference (LSD) analyses at a *p*-value threshold of ≤0.05.

At DOY 286, the total root starch content was 155.56 ± 8.74 mg g^−1^ (DW) in T, 153.14 ± 15.58 mg g^−1^ (DW) in SI, and 127.08 ± 10.87 mg g^−1^ (DW) in S. Figure 7 shows the stained starch area detected by light microscopy and expressed as a percentage of the total section area at DOY 172 and 286. At DOY 172, significant differences were observed between T and SI treatments, with the lowest value in T (21.51%) and the highest value in SI (43.59%). At DOY 286, the values ranged from 38.17% (S) to 43.92% (T) without significant differences between treatments.

## 4. Discussion

In the annual regional reports from ARPA [43], 2022 was indicated as the warmest year for the Piedmont Region in the historical climatic series from 1958 to 2022. In the present work, during 2022, which was also a very dry year in the trial area (522 mL total rainfall detected), both SI and S treatments showed a significant effect in protecting plants from climate stress. Leaf gas exchanges detected in 2022 at different times during the day (Figure 2) showed that in the morning, at comparable levels of stomatal conductance, transpiration and net CO_2_ assimilation were higher in SI and S treatments. Transpiration was significantly higher in SI and S, and net CO_2_ assimilation was significantly higher in S; this could mean that water storage replenishment during the night performed better in SI and S treatments. As a result, stem rehydration was better, and photosynthesis was more active early in the morning in these treatments in comparison to the control plants. In the middle of the day and in the afternoon, when the stress induced by high temperatures is greater, the stomatal conductance also became significantly higher in SI and S treatments, while the net CO_2_ assimilation and the transpiration became significantly higher only in the SI treatments: beyond the better plant water status, this could indicate that the cooling effect of over-tree irrigation protected the photosynthetic apparatus from the excessive temperature [44,45] and also that the possible negative effect of shading on photosynthesis was largely counteracted by the positive effect on the microclimate improvement. Low stomatal conductance, transpiration, and net CO_2_ assimilation associated with a sudden drop of sap flow were previously observed in KiDS-affected plants early in the morning, when transpirative demand rises at sunrise, and they remained low throughout the whole day, indicating a worse water status in comparison to healthy plants [15].

The SI treatment was effective in decreasing the leaf temperature to a level compatible with leaf viability, while in plants without microclimate control, the leaves reached temperatures that cause not only the photosynthesis arrest but also the desiccation (Table 2, Figure 5) [44,45]. Very high leaf temperatures were previously observed in KiDS-affected plants during summer [15].

During 2023, the climate stress was less intense, as evidenced by lower mean temperatures and VPDs (Figure S1) and by lower water volumes needed for irrigation determined by monitoring the soil water depletion with soil moisture sensors. During spring (DOY 136), when over-tree irrigation was still not active, stomatal conductance and net CO_2_ assimilation in T plants were not different from SI and S treatments, while transpiration was lower. During summer (DOY 172), when over-tree irrigation had just begun, transpiration was significantly higher in T than in SI, probably due to a higher need for leaf homeostasis maintenance; net CO_2_ assimilation was significantly lower in S, probably due to shading when not counter-balanced by the additional positive action of over-tree irrigation. In autumn (DOY 286), under not stressed conditions (mean daily temperature = 18.3 °C, VPD = 0.66) and when over-tree irrigation had ceased since 28 days, the SI treatment showed significantly higher values of stomatal conductance, transpiration, and net CO_2_ assimilation in comparison to both T and S, indicating a direct positive effect of over-tree irrigation independently from shading, which continues even when over-tree irrigation was no longer carried out. A positive effect of shading alone was evidenced, nevertheless, by higher values of stomatal conductance and transpiration observed in S in comparison to T. The positive effect of over-tree irrigation for a high until autumn net CO_2_ assimilation can also explain the higher growth, recorded as stem growth diameter, observed in SI plants. The improvement of the canopy microclimate reached by the association of shading and over-tree irrigation induces more favorable conditions for plant physiology; moreover, the direct water delivery to leaves can induce a better water status of the whole plant [46,47]. Indeed, in kiwifruit, the water translocation from roots to leaves can be hindered under high temperature, drought, or VPD stress due to the high vessel vulnerability to embolism and implosion, caused by the wide xylem vessel diameter, numerous large leaves, and anisohydric behavior [3,14,15], inducing the onset of KiDS symptoms [15,48,49,50]. Drip irrigation has largely spread for kiwifruit since the detection of its high vulnerability to root hypoxia and waterlogging. However, the data obtained in this work showed that the water delivered this way was not sufficient to face the plant’s needs: under high transpirative demand, the water delivery by drip irrigation cannot be increased because this would result in waterlogging and then root damages; moreover, water excess in soil promotes the occurrence of soil-borne pathogenic diseases. Consequently, improper irrigation management could have contributed to the onset and spreading of KiDS.

VPD is calculated as the difference between the saturation air vapor pressure and the actual air vapor pressure at a certain temperature; it rises more than proportionally to rising temperatures at a global level, and it is considered an indicator of the air desiccation strength. Plant physiology and anatomy can show signs of adaptation to rising VPD independently from temperature [51]. Hindered hydraulic conductance was observed in KiDS-affected kiwifruit plants under VPD stress, even if soil moisture was maintained at the optimal level [15]. As a result, the direct leaf rehydration achieved with over-tree irrigation can be crucial to survive more intense periods of VPD stress and to prevent the KiDS symptom onset [46,47].

Lower values of SWP were observed in KiDS-affected plants than in healthy plants [15]. During 2023, SWP values did not indicate a water stress status; however, the significantly higher value detected in SI plants at DOY 172, for nearly equal stomatal conductance and net CO_2_ assimilation but lower transpiration, indicated a better water-use efficiency. In autumn, 28 days after the end of over-tree irrigation treatment, SWP rose in T and S, whereas it decreased in SI: this could mean that the plants deprived of over-tree irrigation turned into the same condition as other treatments, where water uptake was possible only from roots, and were then subject to xylem hydraulic conductance restrictions (Figure 4).

Plants from the SI treatment during 2023 showed a gradual increase in stomatal conductance, while they did not show substantial changes in transpiration and net CO_2_ assimilation from DOY 136 to DOY 286, meaning they were broadly balanced (Figure 3). On the other hand, a decrease in leaf gas exchanges was observed in T and S plants from DOY 172 to DOY 286: this could be due to a progressive worsening of plant water status caused by hindered hydraulic conductance, as also evidenced by root xylem vessel characteristics. Indeed, as a long-term adaptation strategy to VPD stress, independently from the water availability in soil, the xylem vessel diameter can decrease to prevent the risk of cavitation, and the vessel density can increase to hold an adequate sap flow [15]. In kiwifruit, the root xylem vessel diameters usually range from 120 to 500 μm [52]; in the present work, narrow root xylem vessel diameters were detected in all treatments (from 57 to 81 μm), indicating a general status of defense from VPD stress in all plants. A narrowing of root xylem vessels was previously observed in the same area and was more accentuated in KiDS-affected plants [14,15] as a response to the trend in rising temperatures and VPDs during the last ten years [53]. The xylem vessel diameter was very low in all treatments in 2023 at DOY 172, probably as a residual effect of the very strong climate stress suffered during the previous year. In the following autumn, at DOY 286, vessel diameter showed an increase in all treatments, but in the SI treatment, it was significantly higher than in T, while it was comparable to T in S plants; this denoted a lower stress status only in SI plants, as confirmed by the highest vulnerability index (Figure 6). Moreover, vessel density was constant from spring to autumn in SI plants, and it was significantly lower in comparison to T and S plants during the summer. Thus, we can postulate that these plants were not under water stress. A higher vessel density of T and S plants was probably a way to increase sap flow when xylem hydraulic conductance was hindered under water stress status [15,51,54].

The positive effect of microclimate control on net CO_2_ assimilation can also explain the higher root starch content observed in SI plants in the summer (Figure 7). Starch in the xylem parenchyma may play a major role in efficient refilling mechanisms, restoring the vessels’ hydraulic conductivity after cavitation [16,17]. Root starch content became similar in all treatments in autumn, when plant growth ceased and starch was stored, probably due to the lower stress on the photosynthetic apparatus and lower transpirative demand in this season; on the other hand, higher starch consumption for the refilling of cavitated vessels during summer can explain the lower content in T. Lower root starch content was observed in KiDS-affected plants [14].

Microclimate control can indirectly affect soil-borne pathogens by altering environmental conditions that influence pathogen survival, growth, and interactions with host plants [20,21,22]; consequently, the reduced risk of waterlogging and the lower temperature reached by over-tree irrigation and shading could also be unfavorable to pathogen development. 

Therefore, it can be stated that the association of over-tree irrigation and shading played a major role in significantly improving plant resilience to face the stresses induced by climate worsening. It could be hypothesized that a higher over-tree water volume or a more frequent delivery during warmer days could have also further improved the plant’s water status under stronger high-temperature stress that occurred in 2022. As a result, microclimate control management could be crucial to prevent the onset of KiDS.

## 5. Conclusions

Irrigation management plays a major role for kiwifruit, due to the high water needs and high root vulnerability to hypoxia and waterlogging. Drip irrigation is considered essential; however, the water delivered this way may be not sufficient to face the plant needs under a climate crisis scenario when temperatures and VPD rise suddenly and remain high for long periods: impaired plant hydraulic conductance hinders water uptake from soil; as a result, increased water delivery by drip irrigation can result in waterlogging, which is detrimental for roots and favorable for the occurrence of soil-borne pathogenic diseases. Consequently, improper irrigation management could have contributed to the onset and spreading of KiDS. Microclimate controlling strategies become necessary following rising plant stress induced by climate change; in this scenario, the association of shading and over-tree irrigation showed to be effective in improving the plant’s physiological status. Shading showed to be not sufficient to protect plants from stress induced by high temperatures and VPDs, while the association of shading and over-tree irrigation significantly improved the plant’s water status and performance: lower leaf temperature throughout the whole vegetative season, higher stem water potential, higher stomatal conductance and photosynthetic activity, and better hydraulic conductance were detected. In order to enhance the use of the water source and avoid wasting it, further studies are suitable to link water delivery to timely plant needs, which should be detected based on plant physiology markers beyond that of environmental parameters such as soil moisture. Matching over-tree irrigation to shading nets could allow for creating the best microclimate conditions, avoiding water waste, and ensuring appropriate responses to plant needs, in order to maintain high yields and plant resilience facing rapidly worsening climate change.

## Figures and Tables

**Table 1:** Location and characteristics of the experimental site.

	Experimental Site Description
Coordinates	N 44.609001, E 7.501853
Area	2625 m^2^
Planting distance	5 m × 3 m
Year of establishment	2020
Cultivar	*Actinidia chinensis* var. *deliciosa* (Hayward)
Training system	T-Bar (pergola)
Shading net	Black-yellow anti-hail shading net
Propagation material	Cuttings
Soil preparation	Soil ridging
Previous crop	Kiwifruit (Hayward)

**Table 2:** Highest leaf temperatures (°C) detected during summer.

	2022		2023	
Treatment	SI	T	SI	T
June	32.5	38.9	35.6	39.0
July	35.1	41.4	35.2	41.7
August	34.1	35.4	35.9	40.9

## Data Availability

The original contributions presented in the study are included in the article.

## Supplementary Materials

The following supporting information can be downloaded at https://www.mdpi.com/article/10.3390/plants14071049/s1: Figure S1. Weather data: (a) daily mean temperature (°C), and (b) vapor pressure deficit, VPD (kPa) recorded during 2022 and 2023 from DOY 135 to DOY 300. Figure S2. Cumulated PAR detected during 2022 from DOY 124 to DOY 304 in T and S (under the net) treatments. Figure S3. Air temperature (°C) and Relative Humidity (%) monitored in T and S (under the net) treatments during 2022 from DOY 124 to DOY 304. Table S1. ANOVA results of data of stomatal conductance (gs), net CO_2_ assimilation (A) and transpiration (E) measured in T, SI and S treatments during 2022 in three different times of the day (morning, midday and afternoon). Table S2. ANOVA results of data of stomatal conductance (gs), net CO_2_ assimilation (A) and transpiration (E) measured in T, SI and S treatments during 2023 in three different times (DOY 136, 172 and 286). Table S3. ANOVA results of data of Stem Water Potential (SWP) measured in T, SI and S treatments during 2023 (DOY 172 and 286). Table S4. ANOVA results of data of xylem vessels diameter, vessel density and vulnerability index measured during 2023 (DOY 172 and 286). Table S5. ANOVA results of data of root starch content (%) measured during 2023 (DOY 172 and 286).
