# Supplementary material for: Orchard Microclimate Control as a Way to Prevent Kiwifruit Decline Syndrome Onset
Source: Plants (Basel). 2025 Mar 28;14(7):1049. doi: 10.3390/plants14071049 (PMC11991025; doi:10.3390/plants14071049)
Supplement: Supplementary file 1 [file plants-14-01049-s001.zip › plants-3538687-supplementary.pdf]

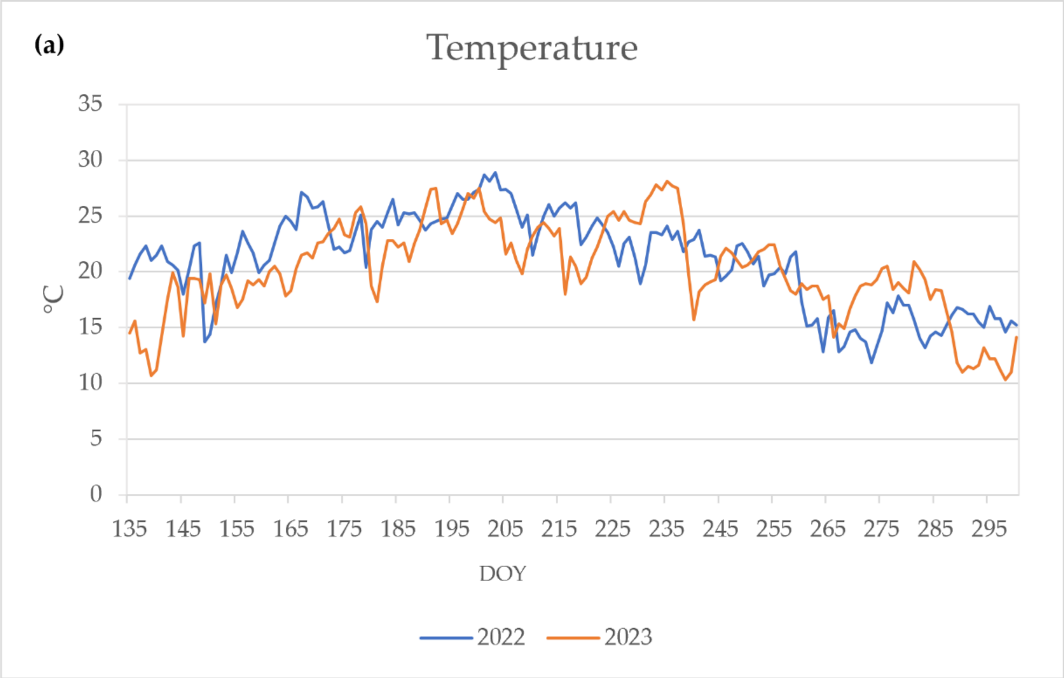

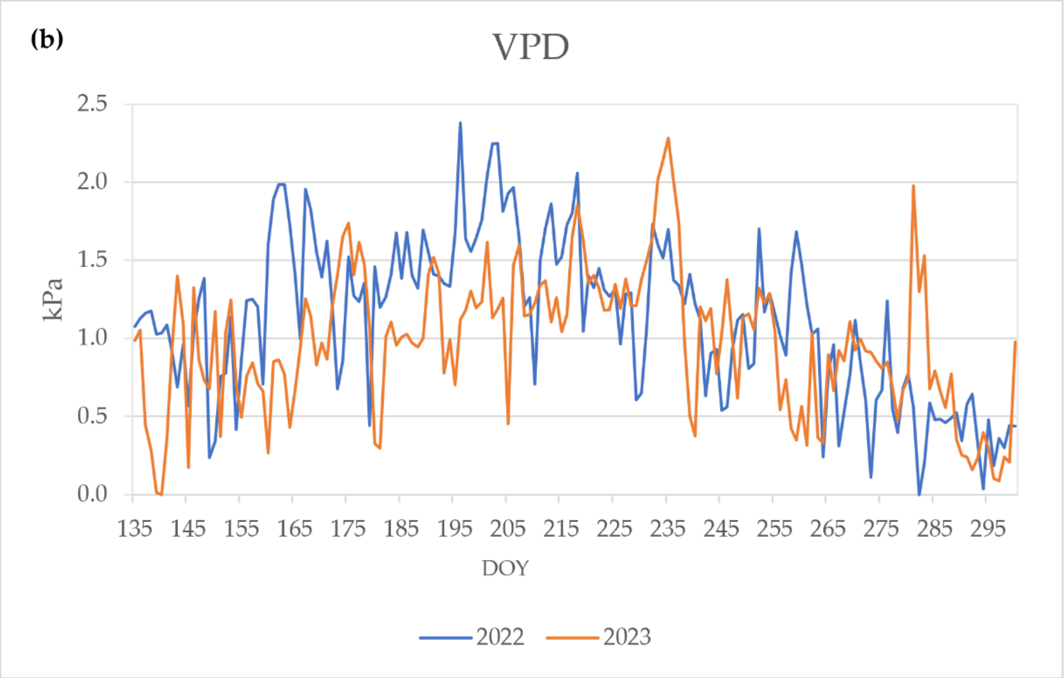

Figure S1. Weather data: (a) daily mean temperature (° C), and (b) vapor pressure deficit, VPD (kPa) recorded during 2022 and 2023 from DOY 135 to DOY 300.

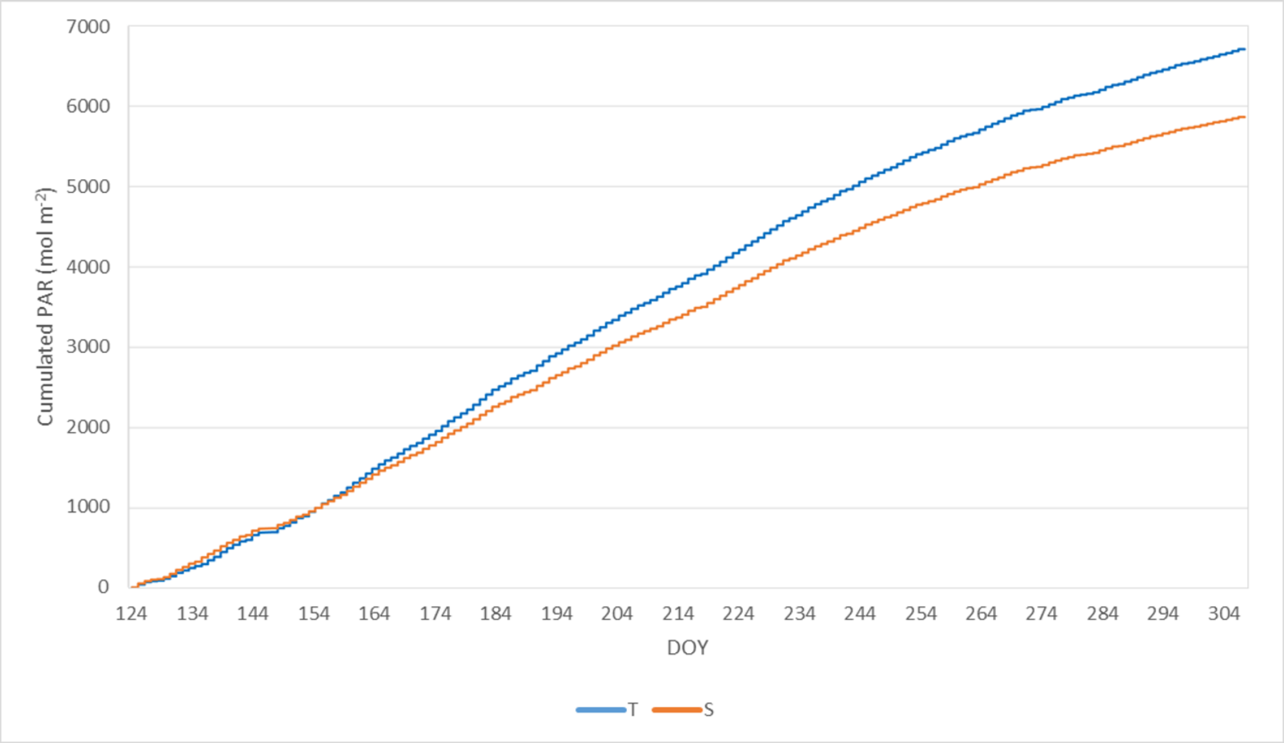

Figure S2. Cumulated PAR detected during 2022 from DOY 124 to DOY 304 in T and S (under the net) treatments.

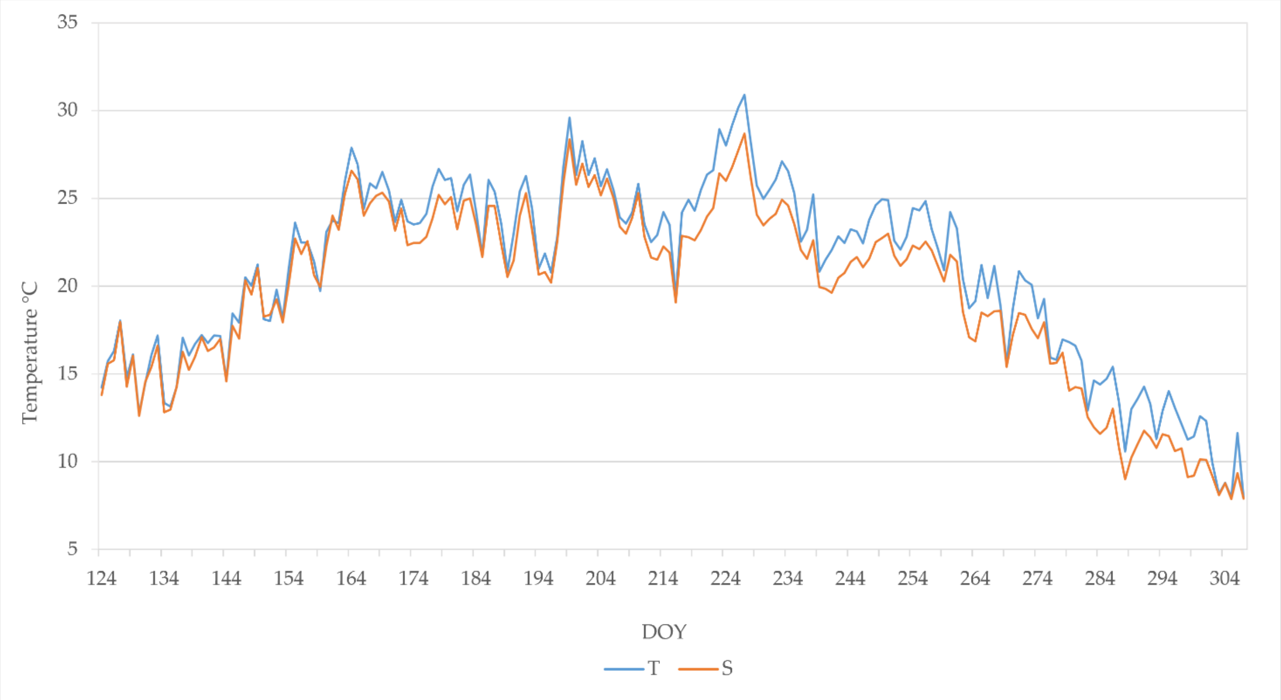

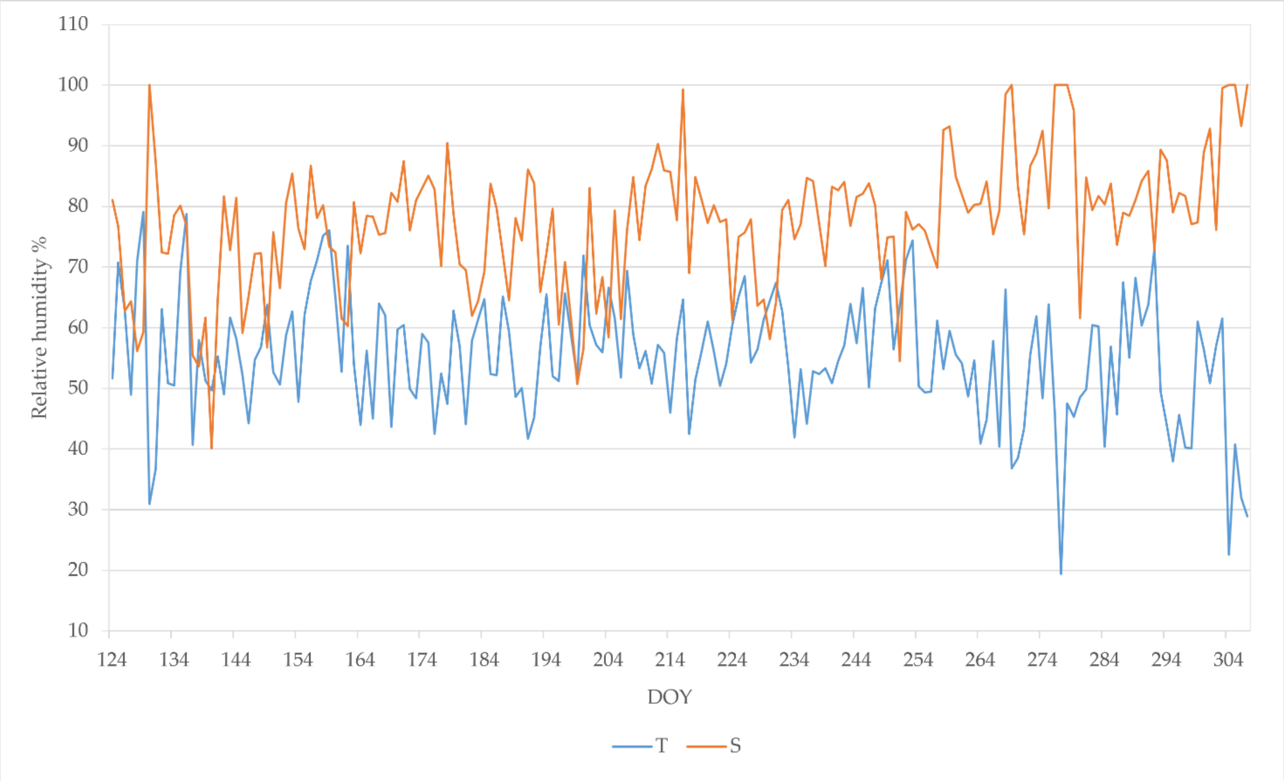

Figure S3. Air temperature (°C) and Relative Humidity (%) monitored in T and S (under the net) treatments during 2022 from DOY 124 to DOY 304.

Table S1. ANOVA results of data of stomatal conductance (gs), net CO_2 assimilation (A) and transpiration (E) measured in T, SI and S treatments during 2022 in three different times of the day (morning, midday and afternoon).

**gs (09.00 a.m.)**	**Sum of sqrs**	**df**	**Mean square**	**F**	**p (same)**
Between groups:	0.01	2	0.00	0.3262	0.7226
Within groups:	0.68	78	0.01		
Total:	0.68	80	0.72		
**A (09:00 a.m.)**	**Sum of sqrs**	**df**	**Mean square**	**F**	**p (same)**
Between groups:	479.80	2	239.90	7.406	0.001136
Within groups:	2526.69	78	32.39		
Total:	3006.48	80	0.00		
**E (09:00 a.m.)**	**Sum of sqrs**	**df**	**Mean square**	**F**	**p (same)**
Between groups:	17.57	2	8.78	10.99	6.23E-05
Within groups:	62.33	78	0.80		
Total:	79.89	80	0.00		

**gs (1:30 p.m.)**	**Sum of sqrs**	**df**	**Mean square**	**F**	**p (same)**
Between groups:	0.04	2	0.02	6.087	0.003496
Within groups:	0.26	78	0.00		
Total:	0.30	80	0.00		
**A (1:30 p.m.)**	**Sum of sqrs**	**df**	**Mean square**	**F**	**p (same)**
Between groups:	305.92	2	152.96	5.94	0.00397
Within groups:	2008.54	78	25.75		
Total:	2314.46	80	0.00		
**E (1:30 p.m.)**	**Sum of sqrs**	**df**	**Mean square**	**F**	**p (same)**
Between groups:	32.14	2	16.07	26.81	1.37E-09
Within groups:	46.75	78	0.60		
Total:	78.88	80	0.00		

**gs (4:30 p.m.)**	**Sum of sqrs**	**df**	**Mean square**	**F**	**p (same)**
Between groups:	0.12	2	0.06	24.55	5.38E-09
Within groups:	0.19	78	0.00		
Total:	0.31	80	0.00		
**A (4:30 p.m.)**	**Sum of sqrs**	**df**	**Mean square**	**F**	**p (same)**
Between groups:	505.38	2	252.69	8.661	0.000401
Within groups:	2275.61	78	29.17		
Total:	2780.99	80	0.00		
**E (4:30 p.m.)**	**Sum of sqrs**	**df**	**Mean square**	**F**	**p (same)**
Between groups:	33.15	2	16.58	17.01	7.39E-07
Within groups:	76.01	78	0.97		
Total:	109.16	80	0.00		

Table S2. ANOVA results of data of stomatal conductance (gs), net CO_2 assimilation (A) and transpiration (E) measured in T, SI and S treatments during 2023 in three different times (DOY 136, 172 and 286).

gs (DOY 136)	Sum of sqrs	df	Mean square	F	p (same)
Between groups:	0.03	2	0.01	4.112	0.02761
Within groups:	0.10	27	0.00		
Total:	0.12	29	0.03		
**A (DOY 136)**	**Sum of sqrs**	**df**	**Mean square**	**F**	**p (same)**
Between groups:	103.22	2	51.61	2	0.1549
Within groups:	696.88	27	25.81		
Total:	800.10	29	0.16		
**E (DOY 136)**	**Sum of sqrs**	**df**	**Mean square**	**F**	**p (same)**
Between groups:	5.96	2	2.98	9.951	0.000579
Within groups:	8.09	27	0.30		
Total:	14.05	29	0.00		

gs (DOY 172)	Sum of sqrs	df	Mean square	F	p (same)
Between groups:	0.06	2	0.03	2.103	0.1416
Within groups:	0.37	27	0.01		
Total:	0.42	29	0.14		
**A (DOY 172)**	**Sum of sqrs**	**df**	**Mean square**	**F**	**p (same)**
Between groups:	563.18	2	281.59	5.974	0.00711
Within groups:	1272.66	27	47.14		
Total:	1835.83	29	0.01		
**E (DOY 172)**	**Sum of sqrs**	**df**	**Mean square**	**F**	**p (same)**
Between groups:	17.20	2	8.60	6.334	0.005552
Within groups:	36.66	27	1.36		
Total:	53.86	29	0.01		

gs (DOY 286)	Sum of sqrs	df	Mean square	F	p (same)
Between groups:	0.447556	2	0.22	26.91	3.73E-07
Within groups:	0.224514	27	0.01		
Total:	0.67207	29	0.00		
**A (DOY 286)**	**Sum of sqrs**	**df**	**Mean square**	**F**	**p (same)**
Between groups:	1750.69	2	875.34	28.99	1.90E-07
Within groups:	815.364	27	30.20		
Total:	2566.05	29	0.00		
**E (DOY 286)**	**Sum of sqrs**	**df**	**Mean square**	**F**	**p (same)**
Between groups:	47.8337	2	23.92	46.37	1.85E-09
Within groups:	13.9266	27	0.52		
Total:	61.7603	29	0.00		

Table S3. ANOVA results of data of Stem Water Potential (SWP) measured in T, SI and S treatments during 2023 (DOY 172 and 286).

**SWP**					
DOY 172					
	Sum of sqrs	df	Mean square	F	p (same)
Between groups:	0.185	2	0.0925	7.4	0.024
Within groups:	0.075	6	0.0125		
Total:	0.26	8	0.02567		
DOY 286					
	Sum of sqrs	df	Mean square	F	p (same)
Between groups:	0.060556	2	0.030278	0.2995	0.7517
Within groups:	0.606667	6	0.101111		
Total:	0.667222	8	0.725		

Table S4. ANOVA results of data of xylem vessels diameter, vessel density and vulnerability index measured during 2023 (DOY 172 and 286).

VESSEL DIAMETER					
DOY 172					
	Sum of sqrs	df	Mean square	F	p (same)
Between groups:	16.5718	2	8.28592	0.142	0.8695
Within groups:	525.043	9	58.3381		
Total:	541.615	11	0.8619		
DOY 286					
	Sum of sqrs	df	Mean square	F	p (same)
Between groups:	442.562	2	221.281	4.469	0.02757
Within groups:	841.824	17	49.5191		
Total:	1284.39	19	0.03055		

VESSEL DENSITY					
DOY 172					
	Sum of sqrs	df	Mean square	F	p (same)
Between groups:	298.5	2	149.25	4.485	0.04453
Within groups:	299.5	9	33.2778		
Total:	598	11	0.05372		
DOY 286					
	Sum of sqrs	df	Mean square	F	p (same)
Between groups:	144.083	2	72.0417	1.598	0.226
Within groups:	946.875	21	45.0893		
Total:	1090.96	23	0.2315		

VULNERABILITY INDEX					
DOY 172					
	Sum of sqrs	df	Mean square	F	p (same)
Between groups:	7.08313	2	3.54156	5.843	0.02363
Within groups:	5.45511	9	0.606123		
Total:	12.5382	11	0.01694		
DOY 286					
	Sum of sqrs	df	Mean square	F	p (same)
Between groups:	15.9649	2	7.98243	3.488	0.04922
Within groups:	48.0638	21	2.28875		
Total:	64.0287	23	0.04802		

Table S5. ANOVA results of data of root starch content (%) measured during 2023 (DOY 172 and 286).

Root starch content (DOY 172)	Sum of sqrs	df	Mean square	F	p (same)
Between groups:	1261.24	2	630.618	7.325	0.008334
Within groups:	1033.05	12	86.0878		
Total:	2294.29	14			0.00973

Root starch content (DOY 286)	Sum of sqrs	df	Mean square	F	p (same)
Between groups:	201.886	2	100.943	0.8558	0.4342
Within groups:	3892.49	33	117.954		
Total:	4094.38	35			0.4372
